# Supplementary material for: Overlooked riverine contributions of dissolved neodymium and hafnium to the Amazon estuary and oceans
Source: Nat Commun. 2023 Jul 12;14:4156. doi: 10.1038/s41467-023-39922-3 (PMC10338505; doi:10.1038/s41467-023-39922-3)
Supplement: Supplementary file 1 — Supplementary Information [file 41467_2023_39922_MOESM1_ESM.pdf]

Supplementary Information for

Overlooked riverine contributions of dissolved neodymium and hafnium to the Amazon estuary and oceans

Antao Xu[1*], Ed Hathorne[1], Georgi Laukert[1, 2, 3], Martin Frank[1]

[1] GEOMAR Helmholtz Centre for Ocean Research Kiel, Kiel, Germany

[2] Department of Oceanography, Dalhousie University, Halifax, Canada

[3] Woods Hole Oceanographic Institution, Woods Hole, USA

*e-mail: axu@geomar.de

This document contains:

• Supplementary Tables 1-4

• Supplementary Figs. 1-7

Supplementary Table 1. Nd concentration ([Nd]) and Nd isotopic composition (ε_{Nd}) of Fe–Mn oxyhydroxides, residual and bulk suspended particulate matter (SPM) from the Amazon River and Pará River.

Diluted reductive solution		Strong reductive solution		Residual SPM		Bulk SPM		Origin
Labile Fe-Mn oxyhydroxides [Nd]	Labile Fe-Mn oxyhydroxides ε_{Nd}	Fe-Mn oxyhydroxides [Nd]	Fe-Mn oxyhydroxides ε_{Nd}	[Nd]	ε_{Nd}	[Nd]	ε_{Nd}	
21.8 ± 2.6	-8.3 ± 0.1	59.3 ± 8.5	-7.9 ± 0.1	157.3 ± 26.0	-11.8 ± 0.03	238.4 ± 32.1	-11.3 ± 0.2	Amazon
34.2 ± 0.8	-10.6 ± 0.02	105.1 ± 2.2	-10.3 ± 0.1	194.1 ± 2.4	-12.7 ± 0.05	333.4 ± 0.5	-12.1 ± 0.02	Pará

Note: The standard deviation (1SD) is calculated for the mean value of 5 samples in the north Amazon transect and 2 samples in the Pará transect. The unit of [Nd] is μmol kg^{-1}. The diluted reductive solution is a mixture of 0.005 M hydroxylamine hydrochloride/1.5% acetic acid/0.03 M Na-EDTA (sodium-ethylenediaminetetraacetate) solution buffered to pH 4 with NaOH (sodium hydroxide), while the concentrations of hydroxylamine hydrochloride and acetic acid in

the strong reductive solution are 10 times higher. Residual SPM represents the SPM after removal of the Fe-Mn oxyhydroxides phases, while the bulk SPM represents the sum of all phases. Further details of SPM sample treatment have been provided in the methods of the main text.

Supplementary Table 2. Average rare earth element and yttrium (REY) concentrations of Fe–Mn oxyhydroxides, residual and bulk suspended particulate matter (SPM) from the Amazon River and Pará River.

Origin	Amazon River				Pará River			
μmol kg^{-1}	Labile Fe-Mn oxyhydroxides	Fe-Mn oxyhydroxides	Residual SPM	Bulk SPM	Labile Fe-Mn oxyhydroxides	Fe-Mn oxyhydroxides	Residual SPM	Bulk SPM
La	15.7 ± 1.9	42.9 ± 6.4	234.9 ± 40.8	293.4 ± 45.2	25.8 ± 0.3	80.3 ± 2.8	303.1 ± 3.7	409.1 ± 0.6
Ce	47.6 ± 5.9	119.3 ± 19.1	433.7 ± 43.3	600.6 ± 55.4	73.8 ± 1.4	228.4 ± 8.5	510.8 ± 2.3	813.0 ± 7.6
Pr	5.2 ± 0.6	13.9 ± 2.0	51.5 ± 8.7	70.6 ± 10.2	8.3 ± 0.1	25.1 ± 0.6	64.3 ± 1.0	97.7 ± 0.2
Nd	21.8 ± 2.6	59.3 ± 8.5	157.3 ± 26.0	238.4 ± 32.1	34.2 ± 0.8	105.1 ± 2.2	194.1 ± 2.4	333.4 ± 0.5
Sm	5.1 ± 0.6	13.8 ± 2.0	27.0 ± 4.2	45.9 ± 5.7	7.7 ± 0.2	23.4 ± 0.5	32.0 ± 0.3	63.0 ± 0.4
Eu	1.1 ± 0.1	3.0 ± 0.4	5.2 ± 0.7	9.4 ± 1.0	1.7 ± 0.02	5.1 ± 0.1	6.1 ± 0.2	12.9 ± 0.02
Gd	4.5 ± 0.5	12.2 ± 1.8	20.7 ± 3.0	37.5 ± 4.4	6.7 ± 0.1	20.5 ± 0.6	23.5 ± 0.3	50.7 ± 0.4
Tb	0.7 ± 0.1	1.8 ± 0.3	3.1 ± 0.4	5.6 ± 0.6	1.0 ± 0.01	3.0 ± 0.1	3.3 ± 0.1	7.3 ± 0.003
Dy	3.5 ± 0.4	9.7 ± 1.4	20.7 ± 2.4	34.0 ± 3.3	5.3 ± 0.05	16.2 ± 0.5	21.5 ± 0.7	43.0 ± 0.1
Y	31.2 ± 3.3	82.0 ± 11.2	236.1 ± 28.2	349.3 ± 34.1	48.6 ± 0.3	137.3 ± 1.1	236.4 ± 9.1	422.2 ± 7.7
Ho	0.6 ± 0.1	1.8 ± 0.3	4.4 ± 0.5	6.7 ± 0.6	1.0 ± 0.01	2.9 ± 0.1	4.5 ± 0.2	8.4 ± 0.1
Er	1.5 ± 0.2	4.3 ± 0.6	13.2 ± 1.5	19.1 ± 1.8	2.4 ± 0.1	7.5 ± 0.2	13.3 ± 0.3	23.2 ± 0.1
Yb	1.1 ± 0.1	3.3 ± 0.5	13.5 ± 1.3	17.9 ± 1.5	1.8 ± 0.02	5.8 ± 0.1	13.6 ± 0.4	21.2 ± 0.4
Lu	0.1 ± 0.02	0.4 ± 0.1	2.0 ± 0.2	2.6 ± 0.2	0.2 ± 0.01	0.8 ± 0.01	2.1 ± 0.1	3.1 ± 0.04

Note: The standard deviation (1SD) is calculated for the mean value of 5 samples in the north Amazon transect and 2 samples in the Pará transect. REY concentration is given in μmol kg^{-1}.

Supplementary Table 3. Calculated estuarine water Nd isotopic composition (ε_{Nd}) changes in the mid- to high-salinity zone of the south Amazon transect resulting from the influence of suspended particulate matter (SPM) from the Amazon River and Pará River.

Sta_ID	Sal [psu]	ε_{Nd} measured	[Nd] measured [pmol kg^{-1}]	[Nd] conservative [pmol kg^{-1}]	ε_{Nd} conservative	[Nd] addition [pmol kg^{-1}]	SPM content [mg L^{-1}]	Labile Fe-Mn oxy. fraction dissolved	ε_{Nd} labile Fe-Mn influenced	Residue fraction dissolved	ε_{Nd} residue influenced	Bulk fraction dissolved	ε_{Nd} bulk influenced	ΔNd_{Fe-Mn}	$\Delta Nd_{residue}$	ΔNd_{bulk}
SPM origin								Amazon River, Pará River								
AM18	9.3	-10.3	26.1	25.8	-9.8	0.3	2.7	0.52%, 0.33%	-9.8, -9.8	0.07%, 0.06%	-9.8, -9.8	0.05%, 0.03%	-9.8, -9.8	-0.4, -0.4	-0.4, -0.4	-0.4, -0.4
AM17	12.3	-10.6	27.6	25.3	-9.9	2.3	3.6	2.83%, 1.80%	-9.9, -9.9	0.39%, 0.32%	-9.9, -9.9	0.26%, 0.19%	-9.9, -9.9	-0.8, -0.8	-0.8, -0.7	-0.8, -0.8
AM16	15.9	-11.0	27.9	24.7	-9.9	3.3	2.5	6.03%, 3.84%	-9.9, -9.9	0.84%, 0.68%	-9.9, -10.0	0.55%, 0.39%	-9.9, -9.9	-1.1, -1.1	-1.0, -1.0	-1.1, -1.0
AM15	20.5	-11.8	29.3	23.8	-10.0	5.5	4.3	5.82%, 3.70%	-9.9, -10.0	0.81%, 0.65%	-10.1, -10.1	0.53%, 0.38%	-10.0, -10.1	-1.9, -1.8	-1.7, -1.7	-1.7, -1.7
AM36	24.9	-13.2	29.1	23.0	-10.1	6.1	7.8	3.56%, 2.26%	-10.0, -10.1	0.49%, 0.40%	-10.2, -10.3	0.33%, 0.23%	-10.2, -10.2	-3.3, -3.1	-3.0, -3.0	-3.1, -3.0
AM14	26.8	-10.8	34.6	22.7	-10.2	11.9	6.9	7.96%, 5.07%	-9.8, -10.3	1.10%, 0.89%	-10.5, -10.7	0.73%, 0.52%	-10.4, -10.6	-1.0, -0.5	-0.2, -0.1	-0.4, -0.2
AM13	30.7	-13.5	32.2	22.0	-10.5	10.2	—	—	-10.1, -10.5	—	-10.7, -10.9	—	-10.6, -10.8	-3.3, -2.9	-2.7, -2.6	-2.8, -2.7

Notes:

(1) The possible influence of SPM dissolution from the Amazon River and Pará River on the variation of ε_{Nd} in estuarine waters were calculated separately.

(2) The conservative Nd concentration ([Nd]conservative) is calculated between the water samples exhibiting maximum removal in the estuary and the seawater endmember.

(3) The conservative ε_{Nd} signature (ε_{Nd} conservative) corresponds to the two-endmember conservative mixing of Amazon freshwater (S: 0.2, [Nd]: 501.9 pmol kg^{-1}, ε_{Nd}: -9.4) and seawater (S: 35.5, [Nd]: 25.8 pmol kg^{-1}, ε_{Nd}: -11.4) in the transect.

(4) [Nd] addition = [Nd]measured – [Nd]conservative.

(5) Dissolution of labile Fe-Mn oxyhydroxides, residual SPM and bulk SPM is calculated by the equation: contribution = ([Nd] addition / (Fe-Mn oxyhydroxides [Nd] or residual SPM [Nd] or bulk SPM [Nd] / SPM content)) * 100 assuming that the [Nd] addition is only supplied via dissolution of the labile Fe-Mn oxyhydroxides or residual SPM or bulk SPM.

(6) ΔNd = ε_{Nd} measured – ε_{Nd} labile Fe-Mn influenced or ε_{Nd} residue influenced or ε_{Nd} bulk influenced.

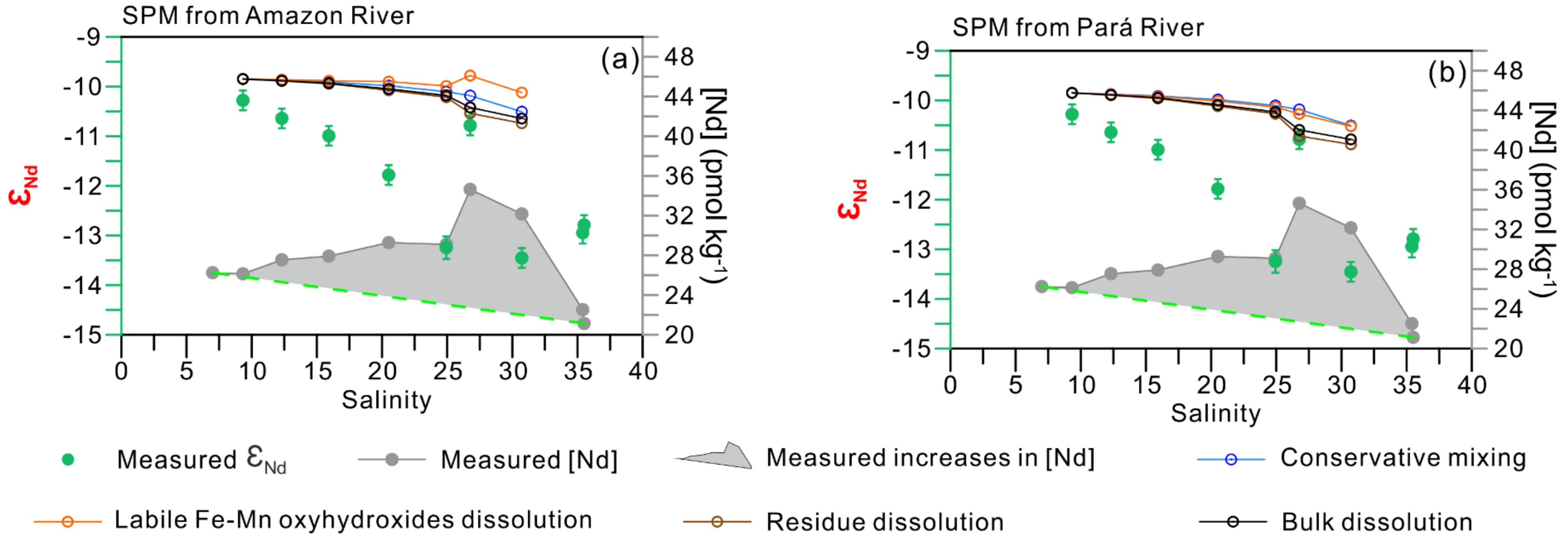

Supplementary Fig. 1. Calculated estuarine water Nd isotopic composition (ε_{Nd}) changes influenced by suspended particulate matter (SPM) dissolution in the mid- to high-salinity zone of the south Amazon transect. The ε_{Nd} changes influenced by the dissolution of labile Fe-Mn oxyhydroxides, residual SPM and bulk SPM from the Amazon River (a) and Pará River (b). The large difference between the modeled ε_{Nd} changes and measured ε_{Nd} in the mid- to high-salinity zone precludes SPM

dissolution as the main source for the increase of [Nd] and less radiogenic ε_{Nd} signatures in the Amazon estuarine plume. Error bars correspond to the 2 standard deviations (2SD) of the ε_{Nd} measurements.

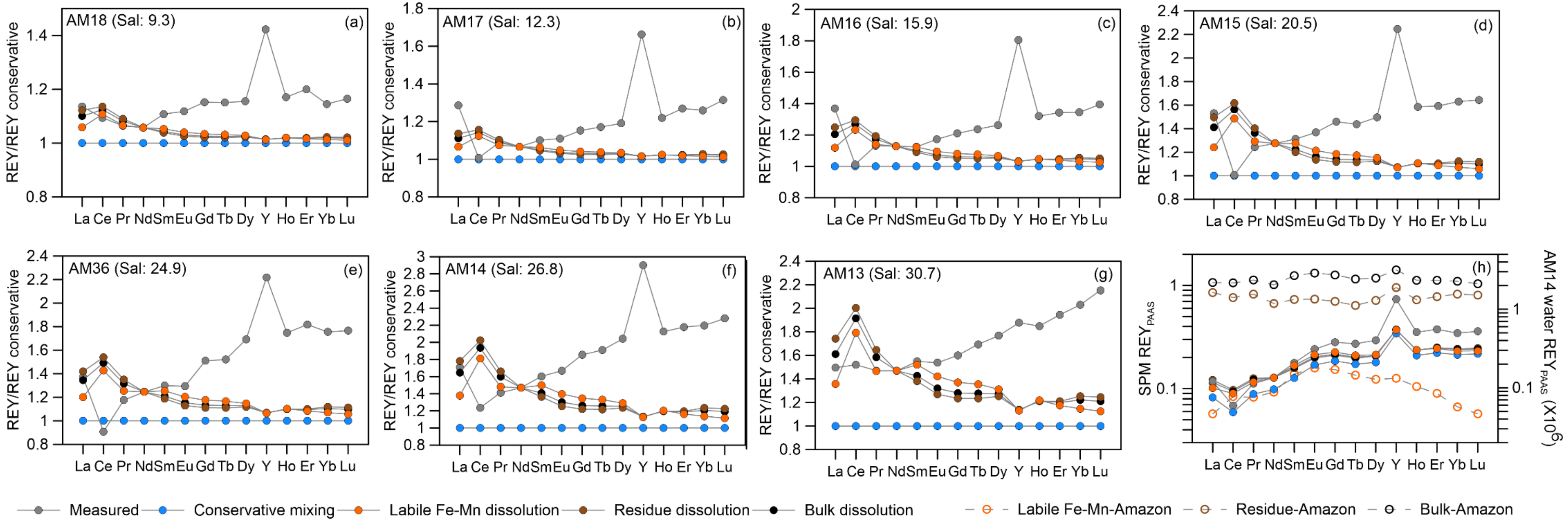

Supplementary Fig. 2. Calculated estuarine water rare earth element and yttrium (REY) changes influenced by suspended particulate matter (SPM) dissolution in the mid- to high-salinity zone of the south Amazon transect. Panels a) to g) show the REY concentrations changes (normalized to conservative mixing) in estuarine water influenced by the dissolution of different phases of SPM from the Amazon River. Panel h) display the estuarine water REY patterns of changes influenced by the dissolution of labile Fe-Mn oxyhydroxides, residual SPM and bulk SPM from the Amazon River. The sample with the highest REY concentrations corresponding to a [Nd] of 34.6 pmol kg^{-1} in the mid- to high-salinity zone of the south Amazon transect is selected to calculate the influence of SPM dissolution on the REY pattern of estuarine water. Panel h) also show the REY patterns of labile Fe-Mn oxyhydroxides, residual SPM and bulk SPM from the Amazon River.

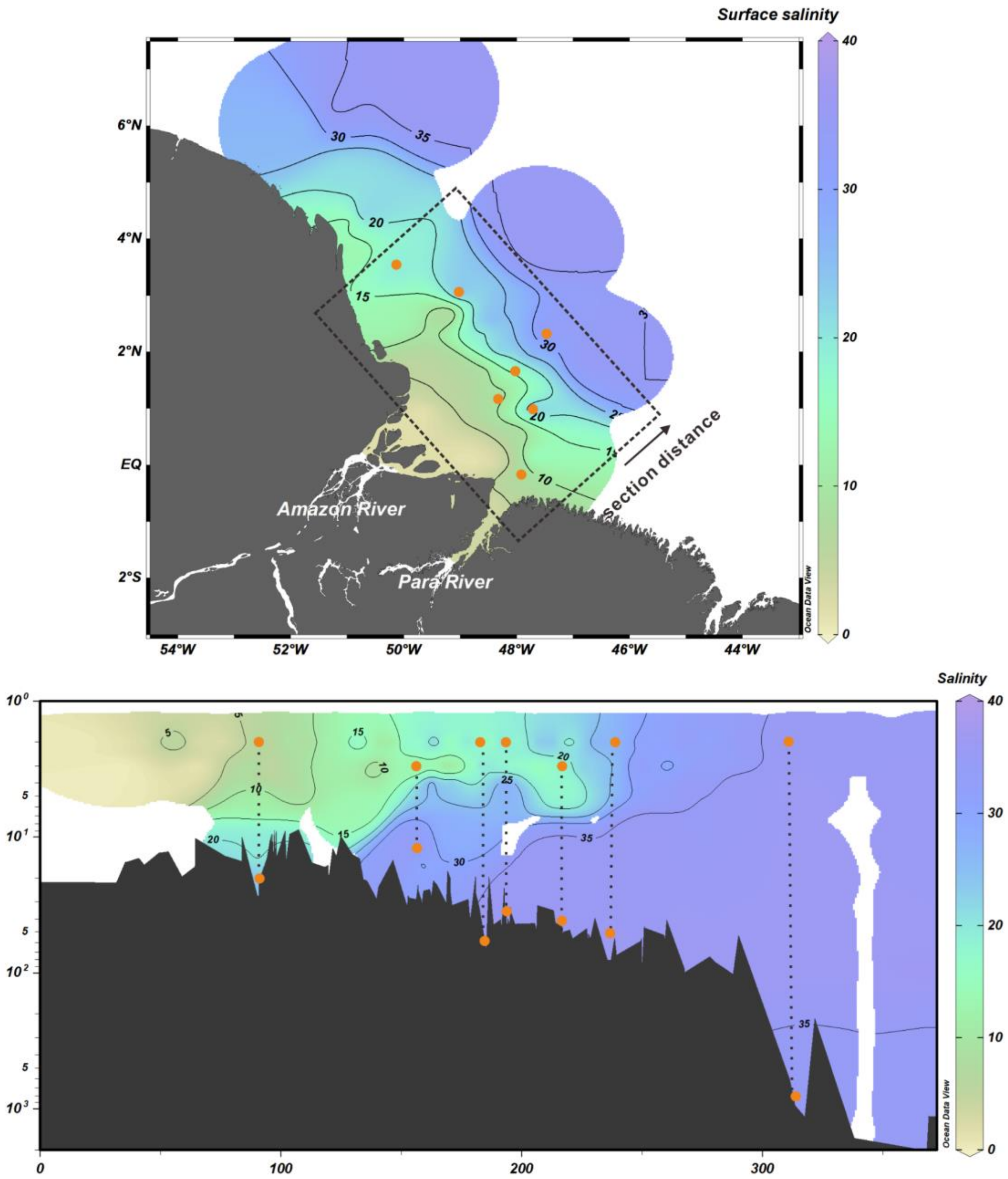

Supplementary Fig. 3. Stations of near-bottom water sampling above the continental shelf area of the Amazon estuary. Salinity is given in psu.

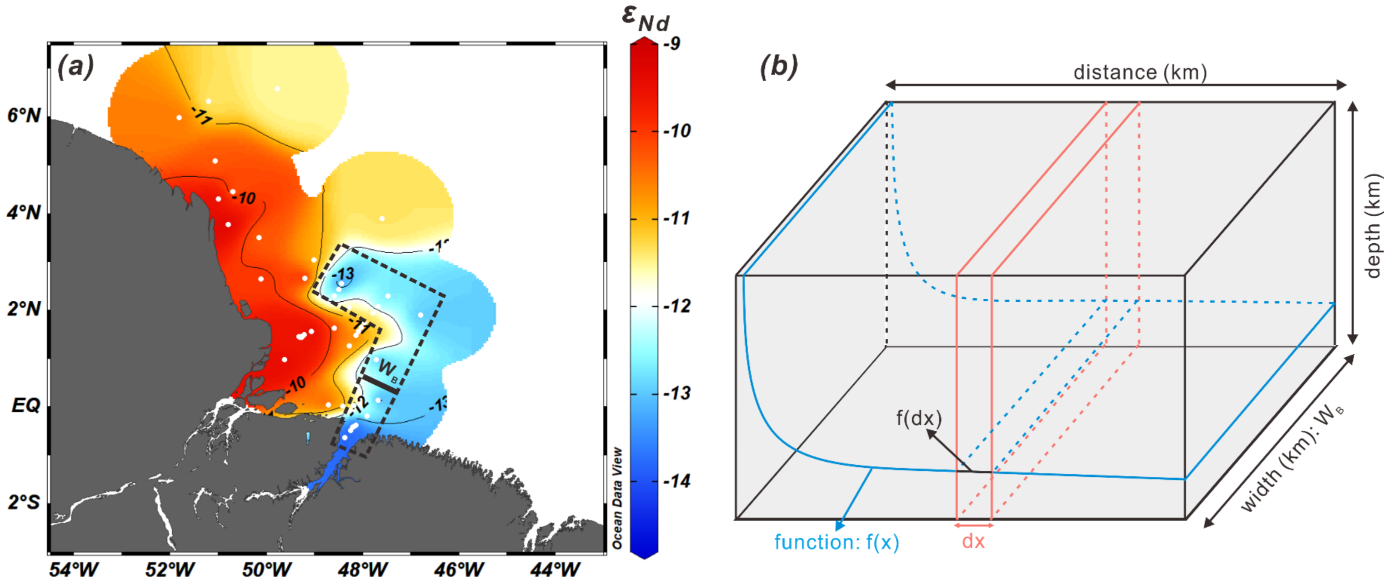

Supplementary Fig. 4. Establishment of a box model for the Amazon estuary. The box includes the Pará estuary and outer Amazon estuary with width W_B, as shown in panel a). The schematic of the Nd and Hf mass calculations in the box model is shown in panel b.

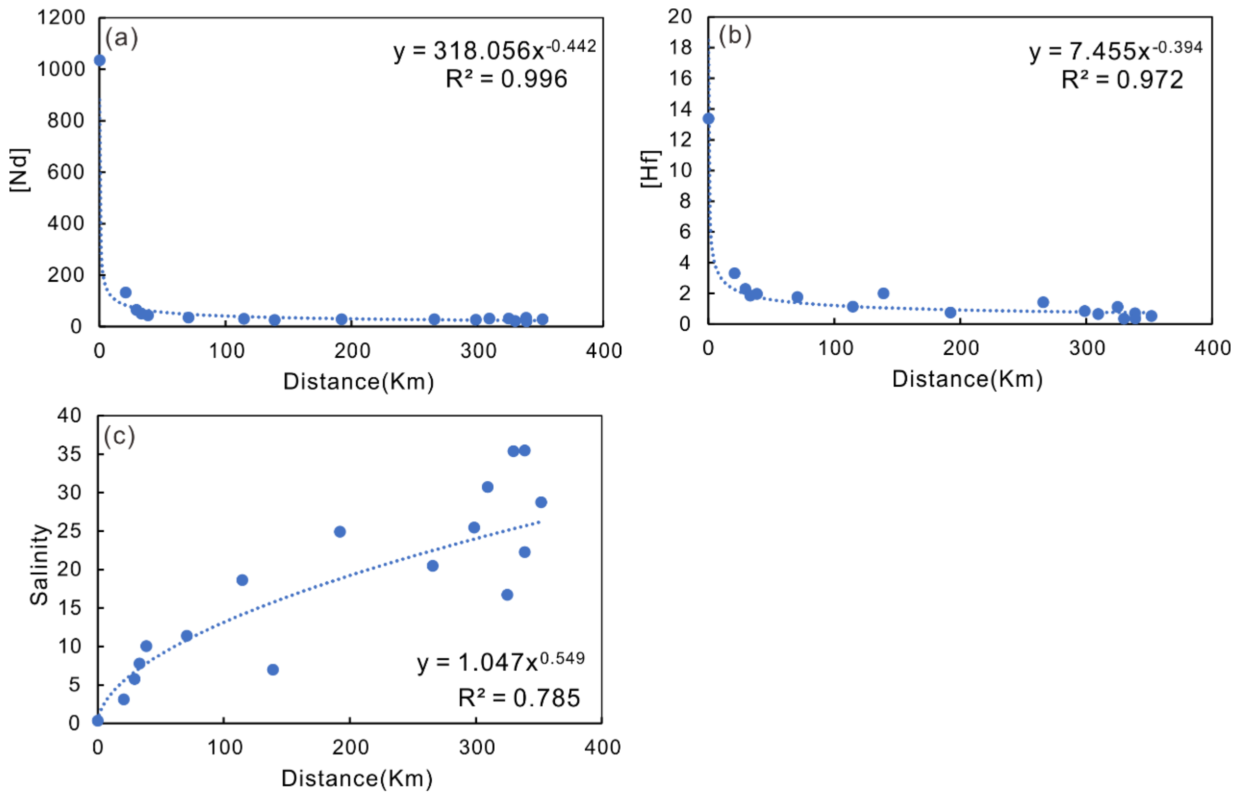

Supplementary Fig. 5. Relationships between the dissolved Nd and Hf concentrations (a and b) and salinity (c) with distance from the Pará River mouth.

Supplementary Table 4. Parameters and results of the box model used in this study.

Parameters or results	Pará River	Amazon River	Seawater
	Box	Box	
[Nd], [Hf] (pmol kg^{-1})	1035.6, 13.4	501.9, 12.3	25.8, 0.4
ε_{Nd}, ε_{Hf}	-14.0, -4.1	-9.4, 1.8	-11.4, -1.0
Salinity	0.4	0.2	35.5
Distance, width and depth (km)	352, 70, 0.005	352, 70, 0.005	
Discharge in May (m^3 day^{-1})	2.6×10^9	2.3×10^{10}	
Seawater volume (m^3)	5.7×10^{10}	5.7×10^{10}	
Freshwater volume (m^3)	6.5×10^{10}	7.5×10^9	
Nd, Hf mass (mol)	5.2×10^3, 1.5×10^2	5.2×10^3, 1.5×10^2	
Nd, Hf maximum removal percentage (%)	95.0%, 82.5%	90.8%, 87.3%	
Gross Nd, Hf supply (mol)	6.8×10^4, 8.7×10^2	0-3.8×10^3, 0-93	
Removed Nd, Hf mass (mol)	6.4×10^4, 7.2×10^2	0-3.4×10^3, 0-81	
Net Nd, Hf supply (mol)	3.4×10^3, 1.5×10^2	0-3.5×10^2, 0-12	1.5×10^3, 23
Calculated mean ε_{Nd}, ε_{Hf}	-13.9 ~ -13.7, -4.1 ~ -3.6		

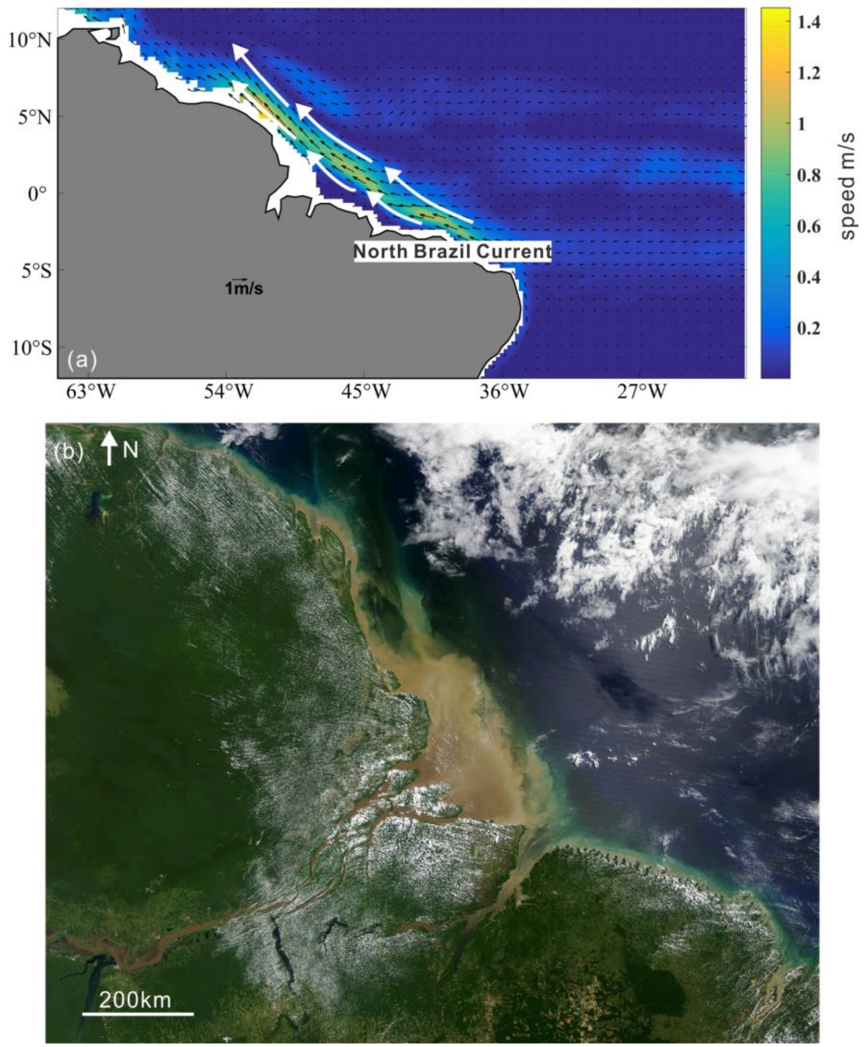

Supplementary Fig. 6. Regional coastal circulation (a)[1] and satellite images of mud distribution in the Amazon estuary (b)[2], documenting the northward deflection of both river plumes. The Amazon plume is usually deflected northward along the coast, while the direct deflection along the coast of the Pará plume is hindered by the strong discharge of the Amazon River. However, further offshore, the highly channeled Pará plume is also deflected to the north by the vigorous North Brazil Current (NBC), which explains the observed higher Pará River fractions at this location.

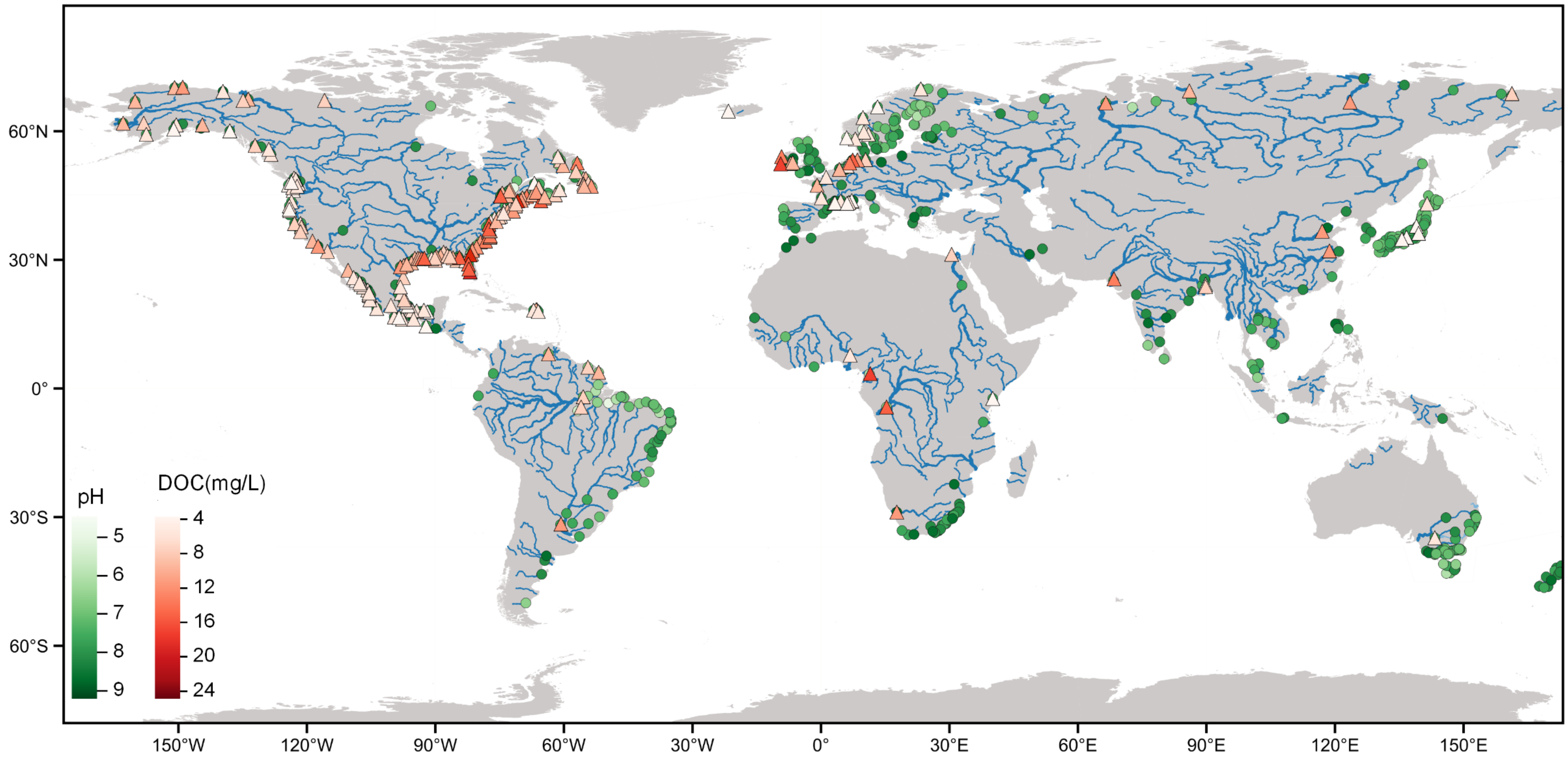

Supplementary Fig. 7. Locations of compiled global river dataset of this study indicated by pH (circles) and dissolved organic carbon concentration ([DOC]) concentration (triangles). Original data for predicting discharge-weighted mean dissolved riverine [Nd] and maximum Nd removal percentage are from GEMStat[3, 4] and GLORICH[5]. Figure produced using QGIS.

Supplementary Discussion

Quantifying uncertainty in the calculation of revised weighted mean riverine dissolved [Nd] using global datasets

In this study, the pH values of 582 global rivers and DOC data of 211 rivers entering the oceans have been compiled to predict [Nd] and maximum removal percentage. To assess the uncertainty in our calculations, we employed measures such as standard deviation (1SD), standard error (1SE), and 95% confidence interval (CI) (listed in Table 2). The calculation of [Nd] yielded a 1SD of 1534 pmol kg^{-1}, confirming a wide range of values in the dataset (n=582), as shown by the positively skewed distribution of [Nd] in global rivers (Fig. 6b). Therefore, we calculated the weighted mean instead of the mean value to reduce the effect of extreme values. It is important to note that the standard deviation primarily represents the statistical dispersion of [Nd] in global rivers. Thus, it was not appropriate to solely rely on the standard deviation for displaying the accuracy and uncertainty of the weighted mean calculation. The small 1SE and narrow range of 95% CI suggest relatively precise estimations of dissolved riverine weighted mean [Nd] and maximum removal percentage, with low levels of uncertainty. Furthermore, it is worth noting that our calculated value of 943 pmol kg^{-1} for the predicted weighted mean dissolved riverine [Nd] is consistent with the estimates of 1054 pmol kg^{-1} by Gaillardet et al. [6] and 894 pmol kg^{-1} by Dang et al. [7]. The close correspondence of our estimate to these previously determined values supports the reliability and accuracy of our calculations.

Methods

Rare earth element and yttrium concentration and neodymium isotope analyses of SPM

The rare earth element and yttrium concentration analyses of Fe–Mn oxyhydroxides (including those treated with a diluted and strong reductive solution) and the residual were measured on an Agilent 7500cx Quadrupole-ICP-MS at GEOMAR. The reproducibility was monitored by repeated measurements of certified reference sediment material MESS-2 (n=3). The REY concentration of bulk SPM is calculated as the sum concentration of Fe–Mn oxyhydroxides and residual SPM. The Nd isotopic composition was measured on a Neptune Plus MC-ICPMS at GEOMAR. Details have been provided in the methods of the main text.

Establishment of the box model

The box model was established following Kaul and Froelich[8]. The relationships between salinity and dissolved Nd and Hf concentrations with distance in the estuary are described by the following equations:

$$\mathrm{F(Nd)} = \mathrm{f}(x) = 318.056 \times x^{-0.442} \quad (1)$$

$$\mathrm{F(Hf)} = \mathrm{f}(x) = 7.455 \times x^{-0.394} \quad (2)$$

$$\mathrm{F(Sal)} = \mathrm{f}(s) = 1.047 \times s^{0.549} \quad (3)$$

The freshwater volume, seawater volume, Nd and Hf masses in the Pará estuary and outer Amazon estuary were calculated by integrating the following equations:

$$\text{Nd or Hf concentration mass } \left(\mathrm{M_{Nd\ or\ Hf-final}}\right) = \int_{0.1}^{\mathrm{distance}} \mathrm{f}(x) \cdot \mathrm{depth} \cdot \mathrm{width} \cdot \mathrm{d}x \ (4)$$

$$\text{Seawater volume } (\mathrm{Vol_{seawater}}) = \int_{0.1}^{\mathrm{distance}} \frac{(\mathrm{f}(s) - \mathrm{Sal_{freshwater}})}{\mathrm{Sal_{seawater}}} \cdot \mathrm{depth} \cdot \mathrm{width} \cdot \mathrm{d}x \ (5)$$

$$\text{Freshwater volume } (\mathrm{Vol_{freshwater}}) = \int_{0.1}^{\mathrm{distance}} \frac{(\mathrm{Sal_{seawater}} - \mathrm{f}(s))}{\mathrm{Sal_{seawater}} - \mathrm{Sal_{freshwater}}} \cdot \mathrm{depth} \cdot \mathrm{width} \cdot \mathrm{d}x \ (6)$$

$\varepsilon_{\mathrm{Nd}}$ or $\varepsilon_{\mathrm{Hf}}$ was calculated by equation (7):

$$\varepsilon_{Nd\ or\ Hf} = \frac{\mathrm{M_{Nd\ or\ Hf-freshwater}} \times \varepsilon_{\mathrm{Nd\ or\ Hf}_{\mathrm{freshwater}}} + \mathrm{M_{Nd\ or\ Hf-seawater}} \times \varepsilon_{\mathrm{Nd\ or\ Hf}_{\mathrm{seawater}}}}{\mathrm{M_{Nd\ or\ Hf-final}} + \mathrm{M_{Nd\ or\ Hf-removal}}} \quad (7)$$

where the $\mathrm{M_{Nd\ or\ Hf\text{-}freshwater\ or\ \text{-}seawater}}$ is the gross Nd or Hf mass supplied by the Amazon River, Pará River or seawater and is calculated by equation (8), $\mathrm{M_{Nd\ or\ Hf\text{-}final}}$ is the Nd or Hf mass in the box calculated by equation (4) and $\mathrm{M_{Nd\ or\ Hf}}$-removal is the Nd or Hf mass removed in the estuary calculated by equation (9):

$$\mathrm{M_{Nd\ or\ Hf}} = \mathrm{Vol_{water}} \times [\mathrm{Nd}] \text{ or } [\mathrm{Hf}] \quad (8)$$

$$\mathrm{M_{Nd\ or\ Hf-removal}} = \mathrm{Vol_{water}} \times [\mathrm{Nd}]_{\mathrm{freshwater}} \text{ or } [\mathrm{Hf}]_{\mathrm{freshwater}} \times \%\ \text{removal} \quad (9)$$

where the % removal reflects the maximum removal percentage of Nd or Hf in the estuary quantified with equation (1) in the main text.

References

1. Laurindo LC, Mariano AJ, Lumpkin R. An improved near-surface velocity climatology for the global ocean from drifter observations. *Deep-Sea Res Part I-Oceanogr Res Pap* **124**, 73-92 (2017).
2. Dauphin L. Mud from the Andes Carried by the Amazon. NASA Earth Observatory https://earthobservatory.nasa.gov/images/147327/mud-from-the-andes-carried-by-the-amazon (2020).
3. United Nations Environment Programme. GEMStat database of the Global Environment Monitoring System for freshwater (GEMS/Water) Programme. International Centre for Water Resources and Global Change K. Accessed 16-11-2020. Available upon request from GEMS/Water Data Centre: gemstat.org, (2017).
4. Virro H, Amatulli G, Kmoch A, Shen L, Uuemaa E. GRQA: Global River Water Quality Archive. *Earth System Science Data* **13**, 5483-5507 (2021).
5. Hartmann J, Lauerwald R, Moosdorf N. GLORICH - Global river chemistry database. PANGAEA, https://doi.org/10.1594/PANGAEA.902360 (2019).
6. Gaillardet J, Viers J, Dupré B. Trace Elements in River Waters. In: *Treatise on Geochemistry* (eds Holland HD, Turekian KK). Pergamon, 225-272 (2003).
7. Dang DH, Wang W, Sikma A, Chatzis A, Mucci A. The contrasting estuarine geochemistry of rare earth elements between ice-covered and ice-free conditions. *Geochim Cosmochim Acta* **317**, 488-506 (2021).
8. Kaul LW, Froelich Jr PN. Modeling estuarine nutrient geochemistry in a simple system. *Geochim Cosmochim Acta* **48**, 1417-1433 (1984).
