# Supplementary material for: Overlooked riverine contributions of dissolved neodymium and hafnium to the Amazon estuary and oceans
Source: Nat Commun. 2023 Jul 12;14:4156. doi: 10.1038/s41467-023-39922-3 (PMC10338505; doi:10.1038/s41467-023-39922-3)
Supplement: Supplementary file 3 — Description of Additional Supplementary Files [file 41467_2023_39922_MOESM3_ESM.pdf]

Description of Additional Supplementary Files

File Name: Supplementary Data 1

Description: This dataset contains the isotope compositions of dissolved neodymium (Nd) and hafnium (Hf), as well as the Nd isotope compositions of suspended particulate matter (SPM). Additionally, it includes the concentrations of rare earth elements and yttrium (REY) and Hf in the surface waters of the Amazon and Pará estuary, as studied.

File Name: Supplementary Data 2

Description: Dataset of pH and organic carbon concentration ([DOC]) of rivers extracted from global datasets of GEMStat and GLORICH.

File Name: Supplementary Information

Description: Information on Supplementary figures and tables.

Note that Supplementary Data 1-2 above are provided in the spreadsheets of "Supplementary Data.xlsx", and Supplementary Information above is provided in the document of "Supplementary Information.pdf".
